# Time-dependent degree of conversion, Martens parameters, and flexural strength of different dual-polymerizing resin composite luting materials

**DOI:** 10.1007/s00784-021-04091-4

**Published:** 2021-08-03

**Authors:** Matthias Kelch, Bogna Stawarczyk, Felicitas Mayinger

**Affiliations:** Department of Prosthetic Dentistry, University Hospital, LMU Munich, Goethestrasse 70, 80336 Munich, Germany

**Keywords:** Dual-polymerizing resin composite luting materials, Raman spectroscopy, *Degree of conversion*, *Martens hardness*, *Elastic indentation modulus*, *Biaxial flexural strength*

## Abstract

**Objective:**

To investigate the degree of conversion (DC), Martens hardness (HM), elastic indentation modulus (E_IT_), and biaxial flexural strength (BFS) of six dual-polymerizing resin composite luting materials initially and after 2 and 7 days of aging.

**Materials and methods:**

Specimens fabricated from Bifix QM (BIF; VOCO), Calibra Ceram (CAL; Dentsply Sirona), DuoCem (DUO; Coltène/Whaledent), G-CEM LinkForce (GCE; GC Europe), PANAVIA V5 (PAN; Kuraray Europe), and Variolink Esthetic DC (VAR; Ivoclar Vivadent) (*n* = 12 per material) were light-polymerized through 1 mm thick discs (Celtra Duo, Dentsply Sirona). DC, HM, and E_IT_ were recorded directly after fabrication, and after 2 and 7 days of aging. As a final test, BFS was measured. Univariate ANOVAs, Kruskal–Wallis, Mann–Whitney *U*, Friedman, and Wilcoxon tests, and Weibull modulus were computed (*p* < 0.05).

**Results:**

While CAL presented low DC, HM, E_IT_, and BFS values, DUO and BIF showed high results. Highest Weibull moduli were observed for VAR and DUO. DC and Martens parameters increased between the initial measurement and 2 days of aging, while aging for 7 days provided no further improvement.

**Conclusions:**

The choice of dual-polymerizing resin composite luting material plays an important role regarding chemical and mechanical properties, especially with patients sensitive to toxicological issues. DUO may be recommended for bonding fixed dental prostheses, as it demonstrated significantly highest and reliable results regarding DC, HM, and BFS. As DC and HM showed an increase in the first 48 h, it may be assumed that the polymerization reaction is not completed directly after initial polymerization, which is of practical importance to dentists and patients.

**Clinical relevance:**

The chemical and mechanical properties of dual-polymerizing resin composite luting materials influence the overall stability and long-term performance of the restoration.

## Introduction

In the case of substance or tooth loss, fixed dental prostheses (FDPs) made of tooth-colored materials (e.g., ceramic or high-performance polymers) or conventional metal alloys represent a common treatment option to restore function and esthetics in the oral cavity [1]. To ensure long-term success, the intraoral fixation of restorations fabricated in the dental laboratory is of paramount importance [2, 3]. The choice of luting agent depends on numerous factors such as the restorative material and the specific clinical situation [4]. While the use of FDPs in the anterior region may require a high translucency on part of the luting material, deep subgingival defects in the posterior region that impede drainage can call for the use of conventional cements [5]. In this extensive field, resin composite luting materials aim to combine outstanding optical and mechanical properties. Advantages include an improved retention and seal of margin, a negligible solubility, and the preservation of dental hard tissue, as macro retentive preparations become obsolete [6]. The development of dual-polymerizing resin composite luting material allows a quick sealing of the restoration margins, which is crucial for clinical settings presenting themselves with a challenging drainage situation [7]. Self-polymerizing initiators aim to enable a comprehensive conversion of the luting agent in situations, where the restoration material is too thick or opaque to allow the transmission of light [8]. An insufficient degree of conversion (DC) can increase the solubility of resin composite luting materials and entail microleakage at the restoration margins [9], which can in the long run cause an undermining formation of caries that can result in the total failure of the restoration [10, 11]. An insufficient polymerization may furthermore reduce the color stability of the luting material and impair the bond strength to natural tooth substances [12, 13]. In addition, a low DC can compromise biocompatibility, an important factor when regarding the ever growing allergic potential of today’s patient cohort [14]. The mechanical properties of resin composite luting materials (e.g., microhardness) are also affected by the achieved DC [15, 16]. With low mechanical properties hampering the overall stability and long-term success of the restoration [2], the present study aimed to investigate the mechanical and chemical properties of different dual-polymerizing resin composite luting materials over time.

For this purpose, the DC, Martens hardness (HM), elastic indentation modulus (E_IT_), and biaxial flexural strength (BFS) of six dual-polymerizing resin composite luting materials with different chemical compositions were examined. The aim of this investigation was to investigate, whether these materials showed different characteristics in the course of time. The tested hypotheses stated that neither the use of different resin composite luting materials nor the aging interval showed an impact on DC, HM, E_IT_, or BFS.

## Materials and methods

**Fig. 1:**
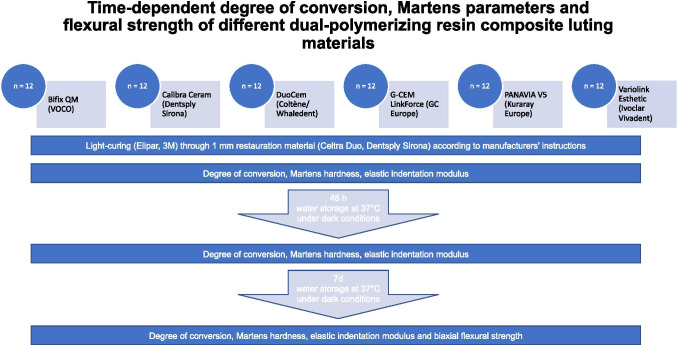
Study design

DC, HM, E_IT_, and BFS of six different *dual-polymerizing* resin composite luting materials were examined initially, after 2 days and after 7 days of aging (Fig. 1, Table 1).

**Table 1:** Material, abbreviation, manufacturer, LOT. no, processing guidelines, and composition of the resin composite luting materials used

Material	Abbreviation	Manufacturer	LOT.no	Processing guidelines	Chemical composition*
Bifix QM	BIF	VOCO, Cuxhaven, Germany	1,938,109	20 s light-polymerization	10–25% Bis-GMA, 10–25% 1,6-hexanediylbismethacrylate, ≤ 2.5% catalyst
Calibra Ceram	CAL	Dentsply Sirona, Charlotte, USA	00,023,656	20 s light-polymerization	2.5– < 10% PENTA, 2.5– < 10% UDMA, 2.5– < 10% urethane-modified Bis-GMA dimethacrylate resin, 2.5– < 10% TMPTMA, 2.5– < 10% TEDMA, 0.1– < 1% α,α-dimethylbenzyl hydroperoxide, 0.1– < 1% acrylic acid
DuoCem	DUO	Coltène/Whaledent, Altstätten, Switzerland	J35265	20 s light-polymerization	10– < 15% Bis-GMA, 5– < 10% TEGDMA, 1– < 5% coated zinc oxide, < 1% dibenzoyl peroxide, benzoyl peroxide, < 1% sodium fluoride
G-CEM Linkforce	GCE	GC Europe, Leuven Belgium	1,908,081	20 s light-polymerization	Paste A: 20–25% UDMA, 5–10% dimethacrylate, < 0.5% stabilizer; Paste B: 20–25% UDMA, 5–10% Bis-EMA, 5–10% dimethacrylate, < 1% dibenzoyl peroxide, < 0.5% initiator, < 0.5% BHT
PANAVIA V5	PAN	Kuraray Europe, Okayama, Japan	730,107	10 s light-polymerization	5–15% Bis-GMA, < 5% TEGDMA, silanated barium glass filler, silanated fluoroalminosilicate glass filler, colloidal silica, surface-treated aluminum oxide filler, hydrophobic aromatic dimethacrylate, hydrophilic aliphatic dimethacrylate, dl-Camphorquinone, initiators, accelerators, pigments
Variolink Esthetic DC	VAR	Ivoclar Vivadent, Schaan, Liechtenstein	Y42678	10 s light-polymerization per mm ceramic	10– < 25% ytterbium trifluoride, 3– < 10% UDMA, 3– < 10% 1,10-decandiol dimethacrylate, 1– < 2.5% α,α-dimethylbenzyl hydroperoxide

*As provided by the manufacturer

*Bis-GMA* bisphenol A-glycidyl methacrylate, *PENTA* dipentaerythritol pentaacrylate phosphate, *UDMA* urethane dimethacrylate, *TMPTMA* propylidynetrimethyl trimethacrylate, *TEDMA* triethylene glycol dimethacrylate, *TEGDMA* triethylene glycol dimethacrylate, *Bis-EMA* ethoxylated bisphenol A dimethacrylate, *BHT* butylated hydroxytoluene

### Specimen preparation

Each specimen was fabricated using a hollow acrylonitrile butadiene styrene mold (SD Mechatronik GmbH, Feldkirchen-Westerham, Germany) to create round discs (diameter 12 mm, thickness 1.5 mm). Before injecting the different resin composite materials, the mold was isolated using petroleum jelly (Vaselinum, Fagron GmbH, Barsbüttel, Germany). To simulate clinically relevant results, all luting materials were light-polymerized (Elipar S10, 3 M, Seefeld, Germany) through a silicate ceramic disc of 1 mm thickness. The discs (*n* = 72) were cut from a presintered CAD/CAM blank (Celtra Duo, Shade A2, HT, Dentsply Sirona, Charlotte, USA) using a low-speed diamond saw (Secotom-50 with cutting disc M1D13, Struers, Ballerup, Denmark; rotational speed of 2500 rpm, feed speed of 0.05 mm/s) under constant water cooling. Afterwards, the discs were sintered (LHT 02/16, Nabertherm GmbH, Lilienthal, Germany) according to the manufacturers’ instructions. For each resin composite luting material, polymerization was performed one by one and from one side according to the manufacturers’ recommendations (Table 1) using a new silicate ceramic disc. Before further processing, the surface of each specimen was cleaned with 96% ethanol (Otto Fischar GmbH, Saarbrücken, Germany).

### Aging procedures

Measurements were conducted at 3 different aging intervals:

i) Initial, directly after fabrication, dry at room temperature
ii) After 2 days of distilled water storage in an incubator (HeraCell 150, Heraeus, Hanau, Germany) at 37 °C under dark conditions
iii) After 7 days of distilled water storage in an incubator (HeraCell 150) at 37 °C under dark conditions.

### Measurement of the degree of conversion

**Fig. 2:**
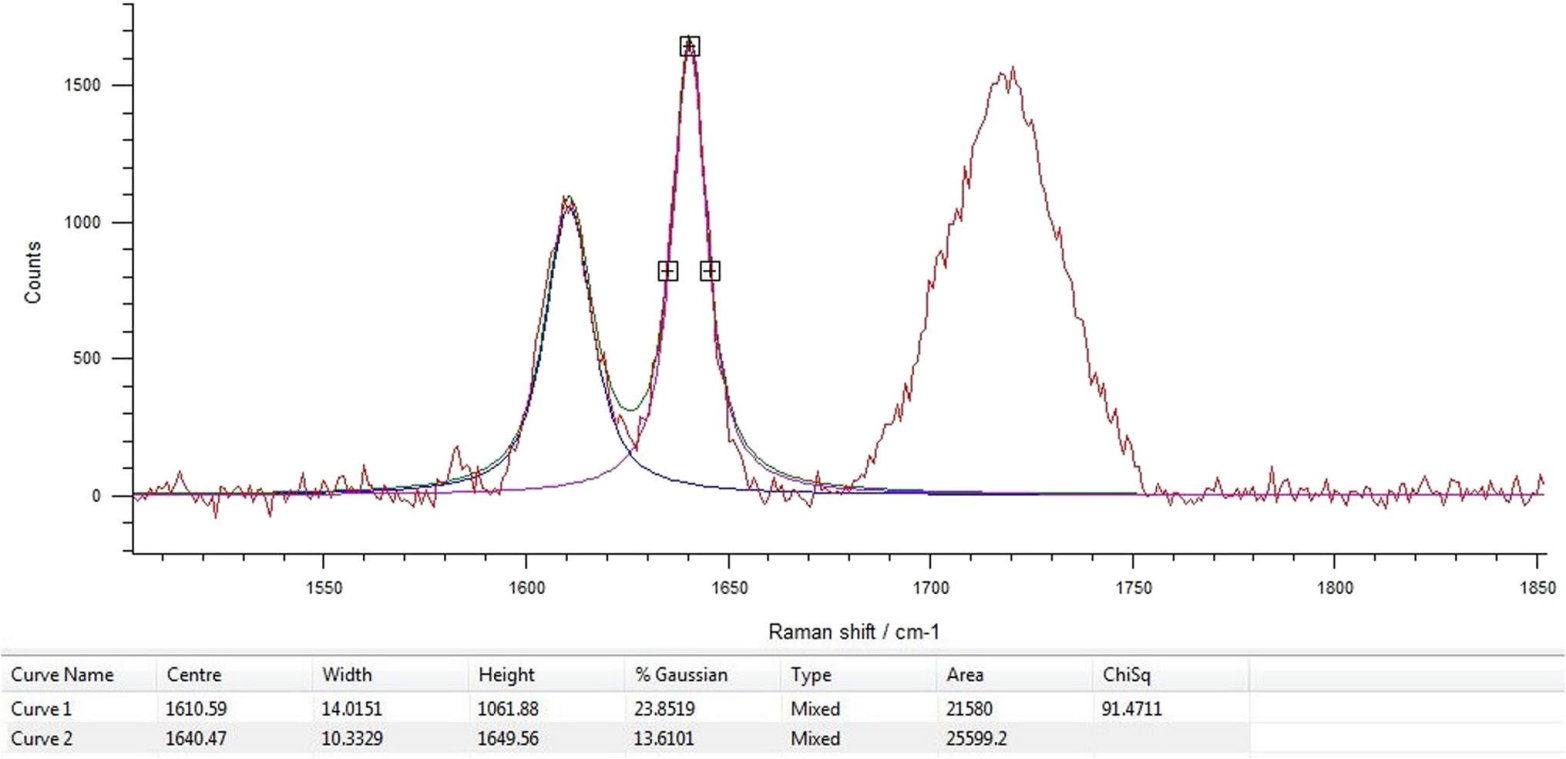
Raman spectrum with peaks at 1610 cm^−1^ and 1640 cm^−1^ (DuoCem, Coltene/Whaledent AG)

DC was determined using a Raman spectrophotometer (inVia Qontor, Renishaw, New Mills, UK). In the first step, the unpolymerized resin composite luting materials were directly applied on a microscope slide to record the Raman scattering of the unpolymerized material (R_unpolymerized_). Ten measurements were performed for each material to obtain an average value for R_unpolymerized_. Raman spectra of the light-polymerized specimens (R_polymerized_) were recorded at all aging intervals. The single mode laser operated at a wavelength of 785 nm. After calibration of the system, Raman scattering was measured with 100% laser power and an irradiation time of 10 s. The obtained data were processed using WiRE 4.4 software (Renishaw). Band heights at peaks 1610 cm^−1^ and 1640 cm^−1^ were automatically determined by the software using curve fit function (Fig. 2). DC was calculated as follows:

$$\mathrm{DC}\left(\%\right)={100}^{*}\left[1-\frac{{R}_{\mathrm{polymerized}}}{{R}_{\mathrm{unpolymerized}}}\right], \mathrm{where }R=\frac{{\mathrm{band height at }1640\mathrm{ cm}}^{-1}}{{\mathrm{band height at }1610\mathrm{ cm}}^{-1}}.$$

### Measurement of Martens hardness and elastic indentation modulus

HM and E_IT_ were determined at each aging interval using the universal testing machine (Zwick/Roell ZHU 0.2, ZwickRoell, Ulm, Germany). The mounted diamond indenter (Vickers pyramid) was vertically pressed into the specimens’ surface. All specimens were loaded with 9.8 N for 2 s. HM and E_IT_ values were determined at three different points per specimen per aging interval (TestXpert v.12.3 Master, ZwickRoell) with the following equations [17]:

$$\mathrm{HM}=\frac{F}{{A}_{S}\left(h\right)}$$

where HM is the Martens hardness, *F* is the test force [N]; *A*_S_ (*h*) is the area of the indenter penetrating the surface at the distance h from the tip [mm^2^] and

$${{E}_{\mathrm{IT}}=\left(1-{v}_{S}^{2}\right)\left(\frac{\sqrt[2]{{A}_{\mathrm{p}}\left({h}_{\mathrm{c}}\right)}}{\sqrt{\pi S}}-\frac{\left(1-{v}_{\mathrm{i}}^{2}\right)}{{E}_{\mathrm{i}}}\right)}^{-1}$$

where *E*_i_ is the elastic modulus of the indenter [N/mm^2^], *A*_p_(*h*_c_) the projected contact area under load [mm^2^], $v$_s_ is the Poisson’s ratio of the specimen with $v$_s_ = 0.4 [18], $v$_i_ is the Poisson’s ratio of the indenter with $v$_i_ = 0.3, and *S* the contact stiffness evaluated from the force removal curve [19].

### Measurement of flexural strength

At the final aging interval, specimens were subjected to a biaxial flexural strength measurement at a room temperature of 23 °C using the universal testing machine (Zwick/Roell Z010, Zwick/Roell, Ulm, Germany) with a load cell capacity of 250 N. The thickness of each specimen was determined with a digital micrometer screw with an accuracy of ± 4 µm (IP65, Mitutoyo Deutschland, Neuss, Germany) prior to placement on a jig with a piston on three balls design. The tempered steel balls forming an equilateral triangle had a diameter of 3.2 mm. Loading with a crosshead speed of 1 mm/min was applied with a 1.6 mm diameter plunger in the center of each specimen until failure. Biaxial flexural strength was calculated using two decimal places and the following formula [20]:

$$\sigma =0.2387{P}^{*}\left(X-Y\right)/{d}^{2}$$

where *σ* is BFS, *P* is fracture load [N], *d* is specimen thickness [mm], and the coefficients *X* and *Y*:

$$\begin{array}{c}{{X=\left(1+\upsilon \right)\mathrm{ln}\left[\left(r2/r3\right)\right]}^{2}+\left[\left(1-\upsilon \right)/2\right]\left(r2/r3\right)}^{2}\\ {Y=\left(1+\upsilon \right)[1+\mathrm{ln}\left[{\left(r1/r3\right)}^{2}\right]+\left(1-\upsilon \right)\left(r1/r3\right)}^{2}\end{array}$$

with *υ* is Poisson’s ratio (*υ* = 0.4), *r*_1_ is the radius of the support circle formed by the three tempered steel balls [mm], *r*_2_ is the radius of the loaded area [mm], and *r*_3_ is the specimen radius [mm].

### Statistical analysis

Descriptive statistics were computed (IBM SPSS Statistics v25.0, IBM Corp, NY, USA). The assumption of normality was tested with the Kolmogorov–Smirnov test. For general analysis, univariate ANOVAs with partial eta squared (*ƞ*_p_^2^) and Scheffé’s post hoc test were computed. To figure out significant differences between the groups’ non-parametric analysis, Kruskal–Wallis, Mann–Whitney *U*, Friedman, and Wilcoxon tests were performed. For a better understanding of the reliability of the tested materials, Weibull modulus was calculated using the maximum likelihood estimation method and 95% confidence interval [21]. For all statistical analyses, *p* < 0.05 were interpreted as statistically significant.

## Results

The results of the descriptive statistics are displayed in Tables 2 and 3. With the Kolmogorov–Smirnov test showing a violation of the assumption of normality for DC (11% not normally distributed), HM (17% not normally distributed), E_IT_ (17% not normally distributed), and BFS (17% not normally distributed), non-parametric tests were carried out. The resin composite luting material showed the highest influence (DC: *p* < 0.001, *η*_p_^2^ = 0.864; HM: *p* < 0.001, *η*_p_^2^ = 0.836; E_IT_: *p* < 0.001, *η*_p_^2^ = 0.634), followed by the aging interval (DC: *p* < 0.001, *η*_p_^2^ = 0.188; HM: *p* < 0.001, *η*_p_^2^ = 0.078; E_IT_: *p* < 0.001, *η*_p_^2^ = 0.065). As the interactions between resin composite luting material and aging showed an impact on HM (*p* = 0.028, *η*_p_^2^ = 0.095) and E_IT_ (*p* = 0.055, *η*_p_^2^ = 0.085), data were analyzed separately according to the tested hypotheses.

**Table 2:** Degree of conversion, Martens hardness [N/mm^2^], and elastic indentation modulus [kN/mm^2^] for all tested groups

	DC		HM		E_IT_	
	Mean (± SD)	95% CI	Mean (± SD)	95% CI	Mean (± SD)	95% CI
i) Initial						
BIF	70.0 ± 2.89^d,A^	[68.0; 71.9]	314 ± 55.6*^d,A^	[277; 349]	7.40 ± 1.74^c,A^	[6.28; 8.51]
CAL	39.9 ± 2.83^a,A^	[37.9; 41.7]	113 ± 28.0^a,A^	[94; 131]	3.38 ± 0.90^a,A^	[2.79; 3.96]
DUO	69.5 ± 2.08^d,A^	[68.0; 70.9]	236 ± 20.7^c,A^	[221; 249]	6.68 ± 1.06^c,A^	[5.99; 7.36]
GCE	71.3 ± 2.35^d,A^	[69.6; 72.8]	220 ± 37.9^c,A^	[194; 244]	5.27 ± 1.60^b,A^	[4.24; 6.29]
PAN	51.6 ± 6.18^b,A^	[47.5; 55.5]	127 ± 24.6^a,A^	[110; 143]	3.87 ± 1.04^ab,A^	[3.19; 4.54]
VAR	58.9 ± 4.23^c,A^	[56.1; 61.7]	149 ± 21.9^b,A^	[134; 164]	4.04 ± 0.89^ab,A^	[3.46; 4.61]
ii) After 2 days						
BIF	73.2 ± 5.32^d,B^	[69.7; 76.6]	377 ± 67.1*^f,B^	[333; 420]	9.04 ± 2.51^d,A^	[7.43; 10.7]
CAL	44.1 ± 4.79^a,B^	[40.9; 47.2]	103 ± 20.9^a,A^	[89; 117]	3.13 ± 0.65^a,A^	[2.70; 3.55]
DUO	72.2 ± 3.18^d,B^	[70.0; 74.2]	270 ± 25.5^e,B^	[252; 287]	7.71 ± 1.55*^d,B^	[6.71; 8.70]
GCE	75.4 ± 2.69^d,B^	[73.6; 77.2]	217 ± 54.3^d,A^	[181; 252]	5.29 ± 1.94*^bc,A^	[4.04; 6.52]
PAN	56.6 ± 5.78^b,B^	[52.8; 60.3]	144 ± 30.0^b,B^	[124; 164]	4.21 ± 1.08^b,A^	[3.51; 4.90]
VAR	64.6 ± 6.08^c,B^	[60.6; 68.5]	178 ± 23.3^c,B^	[161; 193]	5.44 ± 0.82^c,B^	[4.90; 5.97]
iii) After 7 days						
BIF	71.7 ± 3.39^d,B^	[69.4; 73.9]	339 ± 51.1^e,AB^	[305; 372]	7.98 ± 1.90^d,A^	[6.75; 9.19]
CAL	45.1 ± 7.18*^a,B^	[40.4; 49.7]	113 ± 17.1^a,A^	[101; 125]	3.19 ± 0.58^a,A^	[2.80; 3.56]
DUO	72.7 ± 3.48^d,B^	[70.4; 75.0]	269 ± 23.8*^d,B^	[252; 285]	8.28 ± 0.96*^d,B^	[7.65; 8.89]
GCE	75.2 ± 2.11*^d,B^	[73.7; 76.6]	221 ± 42.2^c,A^	[193; 248]	5.23 ± 1.44^bc,A^	[4.30; 6.15]
PAN	57.4 ± 5.47^b,B^	[53.8; 60.9]	150 ± 32.2^b,B^	[128; 171]	4.54 ± 1.39^b,A^	[3.64; 5.43]
VAR	67.3 ± 6.73^c,B^	[62.9; 71.7]	191 ± 15.7^c,B^	[180; 202]	6.06 ± 1.10^c,B^	[5.34; 6.76]

*Not normally distributed

^abc^Different letters present significant differences between resin composite luting materials within one aging interval

^ABC^Different letters present significant differences between aging intervals within one resin composite luting material

**Table 3:** Flexural strength and Weibull moduli for all tested groups

	Flexural strength		Weibull modulus	
	Mean (± SD)	95% CI	*m*	95% CI
BIF	126 ± 48.1^bc^	[95.3; 157]	0.6^a^	[0.2; 1.1]
CAL	79 ± 18.7^a^	[67.0; 90.1]	4.7^bc^	[2.4; 8.6]
DUO	122 ± 11.4^c^	[114; 130]	12.3^d^	[6.7; 22.4]
GCE	125 ± 42.9^bc^	[97.5; 153]	2.5^b^	[1.2; 4.6]
PAN	134 ± 28.8*^c^	[114; 152]	6.1^c^	[3.2; 11.0]
VAR	108 ± 12.2^b^	[99.0; 117]	10.7^ cd^	[5.8; 19.5]

*Not normally distributed

^abc^Different letters present significant differences between resin composite luting materials

**Fig. 3:**
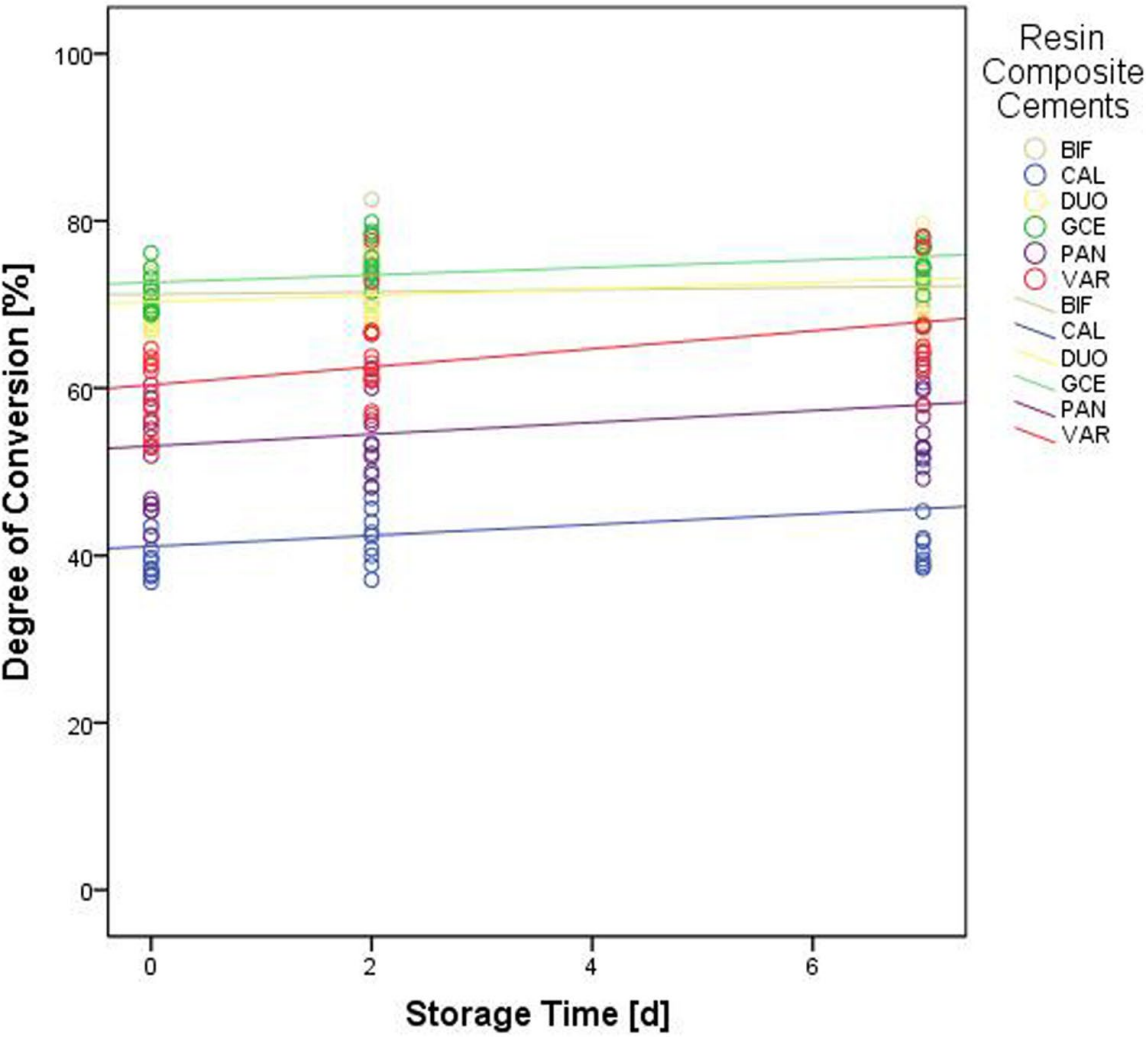
Degree of conversion of the different resin composite luting materials over the course of the three aging intervals

Within one aging interval, CAL showed the lowest DC, followed by PAN and VAR (p < 0.001). DUO, BIF, and GCE presented the highest DC (p < 0.001), with no significant differences being observed between the three materials (p = 0.369–0.999). Values for the DC increased after the initial measurement (p < 0.001), while no difference between aging for 2 or 7 days could be detected (p = 0.767) (Fig. 3).

**Fig. 4:**
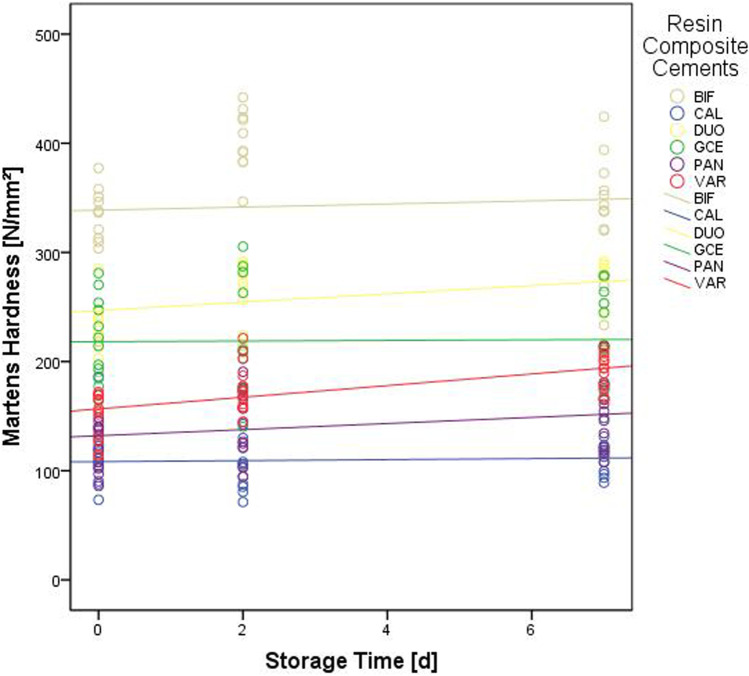
Martens hardness of the different resin composite luting materials over the course of the three aging intervals

**Fig. 5:**
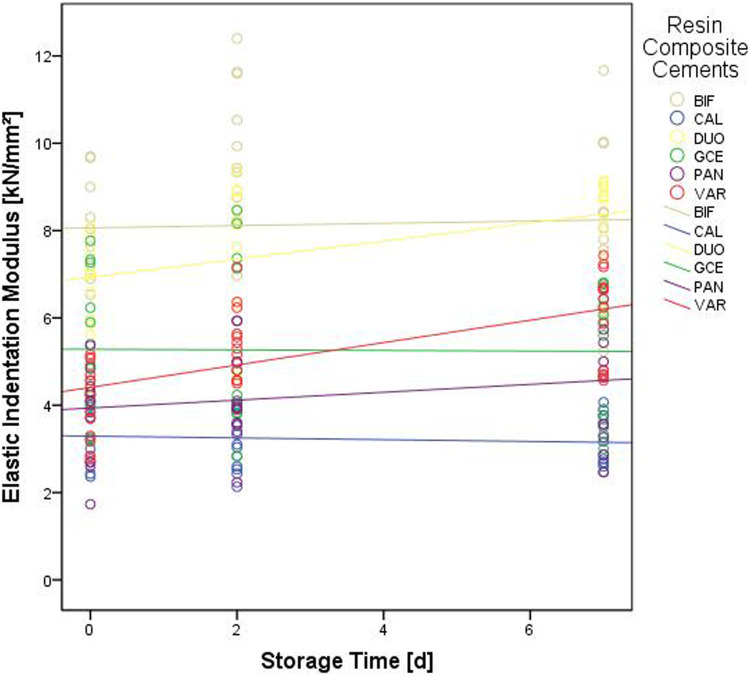
Elastic indentation modulus of the different resin composite luting materials over the course of the three aging intervals

Initially, the lowest HM was observed in the groups CAL and PAN (p < 0.001), while the lowest E_IT_ values were recorded for CAL, PAN, and VAR (p< 0.001). After 2 or 7 days of aging, the lowest values for HM and E_IT_ were found for CAL specimens (p = 0.001–0.017). Regardless of the aging interval, BIF led to the highest HM results (p = 0.001–0.003), while BIF and DUO presented the highest E_IT_ values (p < 0.001). Looking at the effect of aging, an increase in values between the initial measurement and aging for 2 or 7 days was observed for HM values of BIF, DUO, PAN, and VAR (p = 0.001–0.017) and E_IT_ results of DUO and VAR (p=0.001) (Figs. 4 and 5).

The choice of resin composite luting material presented an influence on the BFS, with CAL showing the lowest BFS values, while BIF, DUO, GCE, and PAN demonstrated the highest results (p < 0.001). When regarding the Weibull modulus, the lowest value was observed for BIF, while VAR and DUO presented the highest Weibull modulus.

## Discussion

The aim of this investigation was to examine the DC, HM, E_IT_, and BFS of six dual-polymerizing resin composite luting materials after aging. The tested hypotheses that neither the use of different resin composite luting materials nor the aging interval showed an impact on DC, HM, E_IT_, or BFS had to be rejected.

The polymerization reaction of dual-polymerizing resin composite luting materials, induced by both photo and chemical initiators, leads to an increasing viscosity of the luting material that hinders the movement of the reactive species until conversion comes to a standstill. In the present study, values for the DC of the resin composite luting materials ranged between 39.9 and 75.4. This value range corresponds with previous investigations examining dual-polymerizing resin luting materials with Fourier transform infrared spectroscopy [22, 23]. CAL showed the lowest DC, DUO, and BIF and GCE presented the highest values. With the manufacturers only providing limited information about the chemical composition of the different materials, an interpretation of the influence of the amount and ratio of photo (e.g., camphor quinone) and self-polymerizing initiators (e.g., benzoyl peroxide), their interaction with polymerization inhibitors and fillers, and the composition of the polymer matrix on the results of the present study is complex. Previous investigations employing Fourier transform Raman spectroscopy have reported an impact of the bisphenol A-glycidyl methacrylate (Bis-GMA) and co-monomer content on the degree of conversion [24]. A high DC was observed for the monomer triethylene glycol dimethacrylate (TEGDMA), while Bis-GMA presented significantly lower values [25]. When regarding the contained fillers, it becomes apparent that PAN is the only material where the manufacturer indicated an inclusion of glass fillers in the resin composite luting material’s composition. A positive correlation between a high amount of fillers on the one hand and a low degree of conversion on the other hand has been reported previously [26, 27]. As PAN presented average values by comparison with the examined unfilled materials in the present investigation, future studies will have to examine in how far the filler content plays a crucial part in the DC of dual-polymerizing resin composite luting materials. The duration of light-polymerization may play a negligible role, as CAL presented lower DC values than PAN and VAR, although the duration of light-polymerization was twice as long. As previous examinations did, however, observe an increased light-polymerization time, even above the manufacturers’ instructions, to increase polymerization [28–30], future studies should investigate the influence of varying light-polymerization durations on the degree of conversion of the tested resin composite luting materials. Values for the DC and the Martens parameters increased between the initial measurement and aging for 2 days, while aging for 7 days provided no further improvement. This is in line with previous investigations reporting a high initial increase of the DC immediately after light exposure, followed by a much slower increase in the ensuing hours [31, 32]. When comparing different materials, the level of initial conversion attained from exposure to the polymerization device has furthermore been observed to be a highly influential factor in the final polymerization of light- or dual-polymerized materials [25, 33] 

Although future studies are warranted to confirm the present results, these findings indicate the completion of the polymerization reaction, and in consequence the achieved maximum stability of the luting material and dental restoration, to take up to 48 h. This parameter may be of clinical importance for practicing dentists instructing patients with regard to their postoperative behavior (e.g., avoiding extensive loading in the restored region). It could furthermore alter subsequent treatment procedures, as the present findings clearly indicate an ongoing chemical reaction taking place in the tested dual-polymerizing resin composite luting materials over time. In the case of imperative additional impressions with high-viscosity materials, this procedure should, if feasible, be postponed to a later time, when the polymerization reaction is completed. In line with the observed results for the DC, CAL presented low HM and E_IT_ values, while high values were reported for DUO and BIF. This was to be expected as numerous studies have reported a positive correlation between the DC and the mechanical properties of resin composites [15, 16]. The same trend was seen for the BFS values, where CAL once again presented the lowest values, while DUO, BIF, GCE, and PAN demonstrated the highest results. The computation of the Weibull moduli did, however, yield BIF to present the lowest value, while VAR and DUO presented the highest reliability. While further in vitro and in vivo studies are needed to confirm these results, the present findings indicate the preferred use of resin composite luting material DUO, characterized by a clinically practical light-polymerization duration of 20 s, as it demonstrated significantly highest and reliable results regarding the DC, Martens parameters, and BFS.

The observed results do, however, have to be considered in regard to the limitations of this in vitro investigation, which include a slight variation in the time period between the light-polymerization of each specimen and the measurement of the tested parameters due to the study set-up. Uniform light-polymerization times could allow the elaboration of differences between the tested materials solely based on their composition. In the present study, polymerization was performed through a zirconia reinforced lithium silicate ceramic to imitate the clinical situation as accurately as possible. While this study set-up has been established in numerous previous investigations [23, 34, 35], the choice of ceramic holds a significant impact on the ongoing polymerization reaction, as the ceramic’s crystal structure defines how much light reaches the photo initiators of the resin composite luting material [15, 34]. In this context, the thickness of the employed ceramic also plays a vital part [34]. As in previous investigations, discs of 1 mm thickness were chosen to allow an optimal comparison of the obtained results [15, 23, 35]. Future studies should furthermore examine potential differences in a resin composite luting material’s chemical and mechanical properties following light-polymerization in comparison with self-polymerization. To assess the behavior of resin composite luting materials over time, dynamic testing protocols should be included in future study designs. Clinical investigations should furthermore examine a wider range of ceramic and resin composite luting materials to confirm the results of the present in-vitro investigation. While the determination of the degree of conversion and Martens parameters provides valuable information regarding a material’s biocompatibility and mechanical properties, additional factors such as the esthetic outcome [36], the achieved bond strength [37] and the resulting polymerization stress [38] should be taken into consideration when choosing a resin composite luting material.

## Conclusions

Within the limitations of this study, the following conclusions can be drawn:

1. The dual-polymerizing resin composite luting material DUO may be recommended for luting fixed dental prostheses as it demonstrated significantly highest and reliable results regarding the degree of conversion, Martens parameters, and biaxial flexural strength.
2. As the degree of conversion and the Martens parameters of the tested resin composite luting materials increased between the initial measurement and the simulated clinical condition after 48 h, the completion of the polymerization reaction, which is of practical importance to both dentists and patients, may be deferred.
